# R-Modafinil exerts weak effects on spatial memory acquisition and dentate gyrus synaptic plasticity

**DOI:** 10.1371/journal.pone.0179675

**Published:** 2017-06-23

**Authors:** Bharanidharan Shanmugasundaram, Yogesh D. Aher, Jana Aradska, Marija Ilic, Daniel Daba Feyissa, Predrag Kalaba, Nilima Y. Aher, Vladimir Dragacevic, Babak Saber Marouf, Thierry Langer, Harald H. Sitte, Harald Hoeger, Gert Lubec, Volker Korz

**Affiliations:** 1 Department of Pediatrics, Medical University of Vienna, Vienna, Austria; 2 Department of Pharmaceutical Chemistry, University of Vienna, Vienna, Austria; 3 Centre for Physiology and Pharmacology, Institute of Pharmacology, Medical University of Vienna, Vienna, Austria; 4 Center for Addiction Research and Science, Medical University Vienna, Vienna, Austria; 5 Core Unit of Biomedical Research, Division of Laboratory Animal Science and Genetics, Medical University of Vienna, Himberg, Austria; 6 Neuroproteomics Laboratory, Science Park, Bratislava, Slowakia; Technion Israel Institute of Technology, ISRAEL

## Abstract

Modafinil is a wake promoting drug approved for clinical use and also has cognitive enhancing properties. Its enantiomer R-Modafinil (R-MO) is not well studied in regard to cognitive enhancing properties. Hence we studied its effect in a spatial memory paradigm and its possible effects on dentate gyrus long-term potentiation (DG-LTP). Clinically relevant doses of R-MO, vehicle dimethyl sulfoxide (DMSO) or saline were administered for three days during the hole-board test and in *in vivo* DG-LTP. Synaptic levels of dopamine receptors D1R, D2R, dopamine transporter (DAT), and its phosphorylated form (ph-DAT) in DG tissue 4 h after LTP induction were quantified by western blot analysis. Monoamine reuptake and release assays were performed by using transfected HEK-293 cells. Possible neurotoxic side effects on general behaviour were also studied. R-MO at both doses significantly enhanced spatial reference memory during the last training session and during memory retrieval compared to DMSO vehicle but not when compared to saline treated rats. Similarly, R-MO rescues DG-LTP from impairing effects of DMSO. DMSO reduced memory performance and LTP magnitude when compared to saline treated groups. The synaptic DR1 levels in R-MO groups were significantly decreased compared to DMSO group but were comparable with saline treated animals. We found no effect of R-MO in neurotoxicity tests. Thus, our results support the notion that LTP-like synaptic plasticity processes could be one of the factors contributing to the cognitive enhancing effects of spatial memory traces. D1R may play an important regulatory role in these processes.

## Introduction

Hippocampal LTP is widely considered as a cellular model of memory formation. The underlying molecular machinery has been extensively studied mostly by pharmacological intervention. The role of specific kinases like mitogen activated protein kinase (MAPK) [1], Ca^2+^/calmodulin-dependent protein kinase II (CAMkII) [2] or Protein kinase M zeta (PKM zeta) [3], and neuro-modulatory transmitters like dopamine [4] or noradrenaline [5] in the regulation of LTP and memory have mostly been targeted. Cognition enhancing drugs so far are less well studied. Modafinil (MO) 2-[(diphenylmethyl) sulfinyl] acetamide is a wake-promoting drug approved by the FDA in the year 1998 for treating excessive daytime sleepiness in narcolepsy. Animal model studies revealed that MO has also the potential to improve memory and cognitive abilities including working memory [6], spatial memory [7], fear memory [7], avoidance learning [8], attention [9], impulsive behavior [10], speed of response and accuracy [9].

The underlying mechanisms however are still not understood neither for the wake-promoting properties nor for the cognitive enhancing abilities [11]. The pharmacological target is the dopamine transporter (DAT), which MO inhibits with mediocre affinity [12] and thereby increases the concentration of dopamine in the synaptic cleft [13]. By using *in vitro* binding assays, we previously determined the IC_50_ values for MO, which was 11.11 μM for DAT. The serotonin transporter (SERT) and the noradrenaline transporter (NET) were blocked with even lower affinities, with IC_50_ values of 1547 μM and 182.3 μM, respectively [6]. However, currently, the mode of action of modafinil is believed to rely on an increase of the dopamine concentration in the synaptic cleft which increases cognitive performance [14,15]. This has been found in different brain regions which are critical for cognitive information processing for most of the cognitive enhancers which target the dopaminergic system [16]. However, the mechanisms that are induced by the increase of dopamine are still widely unknown.

It is well known that for chiral compounds, due to the mixture of enantiomers, each racemic form may have different pharmacological properties [17] or different effects on the cognition and behavior of animals [18,19]. Compared to its S-enantiomer, R-Modafinil (R-MO) binds to DAT with approximately three times more affinity [20]. After a single administration, R-MO has higher and long lasting plasma concentrations compared to MO, whereas the half-life is comparable [21].

There is only a small body of literature referring differences in cognitive and behavior effects between MO and R-MO. Brain reward function indicated by intracranial self-stimulation was reduced by MO at a dose of 150 mg/kg body weight which was not found for R-MO [22]. Although there are some studies comparing MO and R-MO regarding their wake promoting effects [23], little is known concerning drug specific effects on cognition. Studies in humans with schizophrenia or HIV positive diagnosis revealed no effect of R-MO on cognitive performance or fatigue [24,25]. However, R-MO can interact with other medications [26,27] which may impede the identification of R-MO specific effects. Animal models and healthy subjects have not been employed and studies on effects upon learning-related synaptic plasticity are still missing. Therefore the aim of the current work was to determine if R-MO has modulating effects on hippocampal *in vivo* LTP and cognitive enhancing effects in a hippocampus-dependent spatial memory paradigm (hole-board test).

## Materials and methods

### Animal housing

Sprague Dawley male rats, aged between 12–14 weeks were used for the neurotoxicity (20 rats), hole-board (40 rats) and electrophysiological (32 rats) experiments. They were bred and maintained in standard Makrolon cages filled with autoclaved woodchips in the Core Unit of Biomedical Research, Division of Laboratory Animal Science and Genetics, Medical University of Vienna. Food and water was available *ad libitum*. Facility conditions were: temperature: 22 ± 2°C; humidity: 55 ± 5%; 12h artificial light/12h dark cycle (light switched on at 7:00am). All procedures were carried out according to the guidelines of the Ethics committee, Medical University of Vienna, and were approved by Federal Ministry of Education, Science and Culture, Austria. Health and wellbeing of the animals were monitored daily. Unexpected deaths of animals did not occur.

### Drug administration

R-MO was chemically synthesized in our laboratory (S1 File, and S1A–S1D Fig). It was freshly dissolved in 100% DMSO and daily injected 30min before the start of the experiments. Rats received intraperitoneally 1ml/kg drug in doses of 1 or 10mg/kg body weight, or the vehicle DMSO or saline.

### Behavioral experiments

#### Hole-board test

Spatial reference memory was assessed using a 1 m × 1 m hole-board maze made of black plastic surrounded by translucent plexiglass wall. Each side of the wall bears proximal spatial cues. Room structures visible outside the board served as distal cues. Four out of sixteen regularly arranged holes (diameter and depth 7 cm) were baited (dustless precision pellets, 45 mg, Bioserv^®^). The pattern of baited holes remained the same during the entire test. Pellets were also present in an area below the board in order to avoid olfactory orientation. Prior to the experiment the rats were familiarized to the experimenter through 15 min handling sessions per day for three days, followed by two days of habituation to the hole-board during which the animals explored the board for 15 min each day with access to food pellets. During this period the weight of each rats were gradually reduced by giving rationed food, so that the rats weight are 85% of its free-feeding body weight before the start of the experiment, in order to increase the motivation to search for food in the maze. Thereafter the rats were trained for three days (five trials on day one, four trials on day 2 and a retention trial at day 3). Every trial lasted for 120 s or until all four pellets were eaten. The apparatus was cleaned with 1% Incidin between trials in order to remove the rat odor cues. The interval between two successive trials for an individual was 20 min. A camera mounted on the room ceiling recorded the performance of the rats in the maze. The hole visits and removals of pellets were noted for each trial. In order to compare rats with similar levels of motivation, rats with less than 40 hole visits in total over the ten trials were excluded from the analysis.

**Fig 1:**
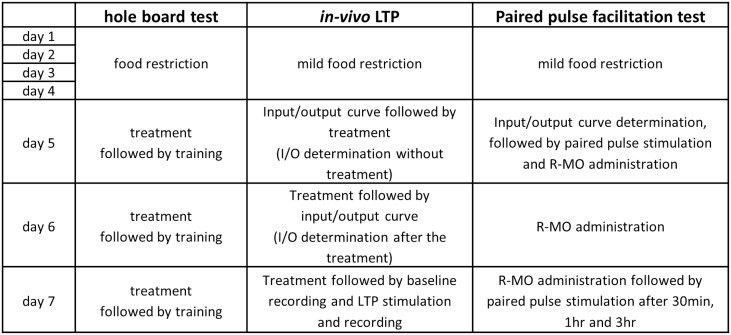
Experimental design of the hole-board and LTP experiments. For *in vivo* LTP and paired pulse facilitation test, treatment frequency and procedure was designed that matches with the hole-board test protocol. The term ‘treatment’ implies that the animals either administered with R-MO or DMSO or saline 30 min before the start of the actual experiment.

Reference memory errors were noted as the number of visits to the unbaited holes. Reference Memory Index (RMI) was calculated using the formula (first + revisits of baited holes)/total visits of all holes. The drug administration and hole-board protocol is presented in Fig 1.

#### Neurotoxicity assay

The drug administration procedure and the sequence are as follows, two days of handling followed by five days of R-MO 10mg/kg i.p. injection. The tests were conducted (one test per day) 30 min after the administration of drug or vehicle in the given order (S1F–S1H Fig). Open field test (measured parameters: total distance moved, resting time, local and large movements, average velocity, number of times crossing the center, frequency of spontaneous changes of direction and time spent in the corners), Elevated plus maze (frequency of leaving the open/closed arm, distance covered, time spent and resting time in each arm), rota rod (the time it can hold in the rotating barrel without falling), Neurological observational battery of tests and forced swim test (total immobility time). The detailed procedure is given in the S1 File.

### *In vivo* LTP

#### Electrode implantation

The rats were implanted with a double recording electrode and a bipolar stimulation electrode. The rats were anesthetized by i.p. application of 50 mg/kg pentobarbital. The head was fixed in a stereotaxic instrument (Kopf Instruments, USA) such that Bregma was 1 mm higher than lambda. The bipolar stimulation electrode was implanted in the right medial perforant path (−6.9 mm AP, 3.5 mm ML, 2.0–2.4 mm DV). The double recording electrode consisted of a pair of insulated stainless-steel wires (125 μm in diameter) of which one was shortened to obtain a length difference of 400 μm between tips. Stereotaxic implantation into the right dentate gyrus (−2.8 mm AP, 1.8 mm ML) yielded optimal signals at a depth of 2.8–3.4 mm. The shorter tip positioned in the laminar molecular layer, recorded the slope (mV/ms) of the negative field excitatory postsynaptic potential (fEPSP) and the longer tip recorded the population spike amplitude (PSA; difference in mV between the first positive peak and the negative deflection of the signal) in the granular cell layer. Electrodes and sockets were fixed with dental cement. Post-surgery the rats were administered subcutaneously with Carprofen (5mg/kg) immediately and 24 hours later to minimize the pain. The animals were given at least 1 week to recover before performing the experiments.

#### LTP recording

The mildly food-deprived rats (overnight starvation) were kept in the recording box prior to the experiment to get acclimatized. A flexible cable connected the electrodes to a swivel, allowing for free movement of the animal. Biphasic constant current pulses (0.1 ms per half wave) were delivered by stimulators (A-M Systems, Isolated Pulse Stimulator, Model 2100). Evoked field potentials were amplified (100×; differential amplifier, A-M Systems, Model 1700, sampled at a rate of 10 kHz, filtered (high pass: 0.1 Hz; low pass: 5 kHz), and converted by an analog-to-digital interface (CED 1401; Cambridge Electronic Design, Cambridge, UK) for storing the data on a computer. fEPSP and PSA were alternatively recorded every 15 min (mean of five stimuli with 10 s inter-stimulus interval). Input/output (I/O) curve were determined twice, with and without treatment, by stimulating with increasing currents (50–400 μA for fEPSP, 50–800 μA for PSA). The details are given in Fig 1. Stimulation intensities were adjusted based on the I/O curve (without treatment) to evoke 40% of the maximal PSA and 60% of the maximal fEPSP, respectively, but never exceeded 400μA. LTP was induced using weak tetanus (three bursts of 15 stimuli, 200 Hz, 0.1 ms pulse duration, 10 s interburst-interval) which was given at the intensity used for PSA recording. In some of the animals only fEPSP or PS could be recorded so that partly the fEPSP and PSA samples are from different animals.

**Paired pulse facilitation test**: In mild food-deprived animals two pulses were paired with intervals of 50, 70, 100 and 150 ms (5 min inter-pair interval) at a stimulation intensity evoking 40% of the maximal PSA or fEPSP. For each interval, the average of three paired pulses (10 s inter-stimulus interval) was calculated. One pulse set was measured prior to R-MO (10mg/kg dose) application, and further measurements took place at 30, 60 min, and 3 h after the application. The details are given in S1I Fig.

#### Dentate gyrus tissue dissection

Immediately after the last recording, i.e. four hours after LTP induction, rats were sacrificed with carbon dioxide asphyxiation and immediately decapitated. The hippocampus tissue was extracted (following a micro-dissection procedure described in the book “Neuroproteomics”, chapter “Dissection of Rodent Brain Regions”[28]) and the dentate gyrus regions were micro dissected under a stereoscope and frozen in -80°C for biochemical analysis.

#### Monoamine neurotransmitter reuptake

Dulbecco’s modified Eagle’s medium, trypsin and fetal calf serum (FCS) were purchased from Sigma-Aldrich Handels GmbH (Austria). [^3^H]5-HT (Hydroxytryptamineceratine sulfate; 5-[1,2-^3^H[N]]; 27.8 Ci/mmol), [^3^H]DA (Dihydroxyphenylethylamine; 3,4-[ring-2,5,6−^3^[H]]-Dopamine; 36.6 Ci/mmol) and [^3^H]MPP^+^ (Methyl-4-phenylpyridinium iodide; 1-[methyl-3H]; 80 Ci/mmol) were purchased from Perkin Elmer, Boston, MA.

HEK293 cells stably expressing human isoforms of DAT, NET and SERT were used for reuptake inhibition assays. All cell lines were seeded on 96-well plates pre-coated with poly-D-lysine (PDL) (5x10^4^ cells/well) 24 h prior to the experiment. Each well was washed with 100 μL of Krebs-HEPES buffer (KHB; 10 mM HEPES, 120 mM NaCl, 3mM KCl, 2 mM CaCl_2_·2H_2_O, 2mM MgCl_2_·6H_2_O, 5 mM D-(+)-Glucose monohydrate, pH 7.3). Cells were pre-incubated 5 min in KHB containing different dilutions (0.01 μM, 0.1 μM, 1 μM, 10 μM, 0.1 mM and 1 mM) of R-MO. R-MO was dissolved first in 99.9% DMSO and subsequently diluted in KHB. Afterwards, cells were incubated in KHB containing same dilutions of R-MO with addition of 0.2 μM [^3^H]-dopamine (for HEK-DAT), 0.05 μM [^3^H]MPP^+^ (for HEK-NET) and 0.4 μM [^3^H]5-HT (for HEK-SERT). Incubation times were 1 min for HEK-DAT and HEK-SERT and 3 min for HEK-NET. For determination of unspecific uptake in HEK-DAT and HEK-NET 10 μM Mazindole were used and 10 μM Paroxetine were used for HEK-SERT. After incubation at room temperature, reactions were stopped by the addition of 100 μL of ice-cold KHB. Finally, cells were lysed with 300 μL of 1% SDS and released radioactivity was measured by a liquid scintillation counter (Tri-carb-2300TR, Perkin Elmer).

#### Dopamine release assay

Monensin sodium salt and D-amphetamine hemisulfate salt were purchased from Sigma-Aldrich Co.

**Fig 2:**
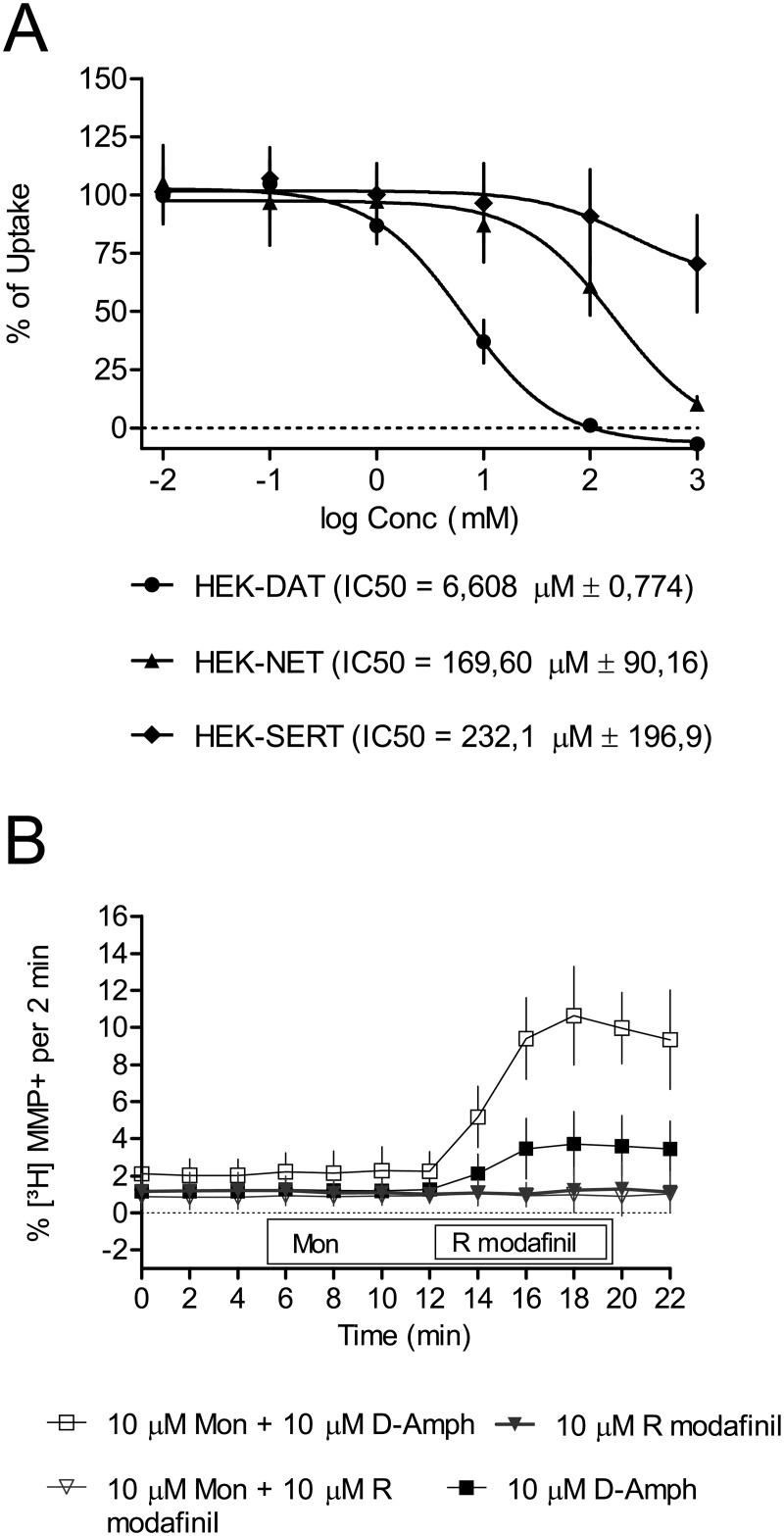
Monoamine neurotransmitter reuptake inhibition and release assay. (A) Inhibition of [^3^H]DA, [^3^H]MPP^+^ and [^3^H]5-HT reuptake by increasing concentrations of R-modafinil in HEK293 cells stably expressing human isoforms of DAT, NET and SERT. Experiments were performed as described in the methods section. Unspecific uptake was determined by using 10 μM mazindole for HEK-DAT and HEK- NET and 10 μM paroxetine for HEK-SERT. The percentage of maximum uptake was determined by using 1% DMSO in KHB. Graph shows mean values ± SD. (B) The release assay was performed in HEK-DAT cells. Cells were grown on PDL-coated coverslips, treated with 0,05 μM [^3^H]MPP^+^ at 37°C for 20 min and washed with KHB for 40 min in superfusion chambers. First three time points (baseline) and next four points were recorded without any compounds and with 10 μM Monensin, respectively. Final five points were with either 10 μM R modafinil or 10 μM D-amphetamine. Non-linear regression analysis was carried out. Values are presented as mean ± SD.

The substrate/efflux experiments were performed as described before by [29]. Briefly, HEK-DAT cells were grown in 5 mm diameter PDL-coated coverslips. Cells were incubated with 0.05 μM [^3^H]MPP^+^ at 37°C for 20 min. The coverslips were transferred onto superfusion chambers (0.2 ml) and excess radioactivity was washed out with KHB for 40 min (0.7 ml/min) at 25°C to obtain stable baselines. The experiment was started with the collection of fractions (2 min) as depicted in Fig 2. During the experiments the buffer was switched either to Monensin or remained at control buffer after the collection of three baseline fractions for another four fractions. Subsequently, R-MO or D-amphetamine was added for another five fractions as indicated in the figure. Finally, the remaining radioactivity was collected by treatment with 1% SDS.

#### Dopamine receptors and transporters level quantification using SDS-PAGE western blotting

The DG tissue was mechanically homogenized with a pipette tip in a Syn-PER synaptic protein extraction reagent (Thermo Scientific, Rockford, IL, USA) containing Protease Inhibitor Cocktail (PIC, Roche Molecular Biochemicals) and Phosphatase Inhibitor Cocktail (PhosStop, Roche Molecular Biochemicals). The homogenate was centrifuged at 1,200 g for 10 min to remove cell debris. The resulting supernatant was centrifuged at 15,000 g for 30 min. The synaptosome pellets were resuspended in solubilization buffer (50mMTrisHCl pH 8.0, 150 mM NaCl, 1% SDS, 1x PhosStop, 1x PIC). Protein concentration was estimated using a bicinchoninic (BCA) assay kit (Pierce, Rockford, IL, USA) according to manufacturer instructions.

Protein levels of D1R, D2R, DAT, and phospho-DAT (ph-DAT) were determined using a standard immunoblotting method. Ten to forty micrograms of each protein sample were incubated at 37°C for 30 min prior to separation on 10% SDS-PAGE. For immunoblotting, proteins were transferred to PVDF membranes (GE Healthcare), blocked for an hour at room temperature with 5% non-fat milk in TBS containing 0.1% Tween-20 (0.1% TBST), and incubated with primary antibodies overnight at 4°C. All antibodies were incubated in 5% non-fat dry milk in 0.1% TBST. Membranes were then washed 6 times for 10 minutes in 0.1% TBST. An HRP conjugated anti-rabbit secondary antibody (1:10000, ab191866, Abcam) was used and blots were visualized with ECL solution (#170–5061, BIO–RAD). Immunoreactive bands were imaged on film, digitized at a resolution of 600 d.p.i. and quantitated using ImageJ software. As loading control membranes were stained with PhastGel Blue-R (GE Healthcare) according to the manufacturer´s instruction. Immunoblot data were normalized to corresponding whole-lane densitometric volumes of total protein-stained membranes as previously described [30]. The specificity of antibodies was determined by [31].

Primary antibodies used in this study: polyclonal rabbit anti-D1R (1∶5000, GeneScript); rabbit anti-D2R (1∶5000, GeneScript), polyclonal rabbit anti-DAT (1∶3000, GeneScript), polyclonal rabbit anti-DATph (1∶3000, GeneScript). Specific peptide sequences were used for immunization and custom-production of the affinity-purified rabbit polyclonal antibodies D1R (-TSTMDEAGLPAERD-), D2R (-NWSRPFNGSEGKAD-), DAT (-TNSTLINPPQTPVEAQERETW-) and ph-DAT (-TNSTLINPPQpTPVEAQERETW-), phosphorylated at T-53. The detailed procedure of synaptosome isolation and western blotting are given in the S1 File.

### Statistical analysis

For the hole-board test RMI measurements, data were analysed for each day seperately. On day 1 and day 2, the effect of training and the effect of drug on learning performance were analysed using a two-way repeated measure ANOVA (Dunnett-T post hoc method) with drug effects as one factor and learning as the second factor. Day 3 retention test data was analysed using univariate ANOVA with Scheffe post hoc test. For LTP analysis two-way repeated measure ANOVA (Dunnett-T post hoc method) was performed for the post-tetanic measurements. For paired pulse facilitation method, two way repeated measure ANOVA with treatement as one factor and inter-pair interval as repeated measure, analysed seperately for different time points. The analysis was done using SPSS 20.0 (IBM) statistics tool.

For neurotoxicity test parameters and western blot densitometry comparison unpaired t-test was used. Non-linear regression analysis was carried out to determine the IC_50_ values for uptake and release assays. These analysis was performed using GraphPad Prism 5.0 tool (GraphPad Software, San Diego, CA, USA). All data are given in mean ± SEM if not stated otherwise. The probability level of p < 0.05 was considered as statistically significant.

## Results

### R-MO blocks DAT but did not facilitate release of dopamine

A reuptake inhibition assay was performed to determine the efficacy of R-MO to block the uptake of substrates [^3^H]DA, [^3^H]MPP^+^ and [^3^H]5-HT by their respective transporters DAT, NET and SERT. R-MO rather selectively targets DAT (IC_50_ = 6.608 ± 0.774 μM; n = 9) as compared to NET (IC_50_ = 169.60 ± 90.16 μM; n = 9) and SERT (IC_50_ = 232.1 ± 196.9 μM; n = 9). Dopamine release assay was done to examine whether R-MO acted as a weak substrate and induces release of [^3^H]MPP^+^ from HEK-DAT cells. 10 μM of R-MO was used while 10 μM D-amphetamine served as a positive control. R-MO does not significantly induced DAT-mediated substrate efflux.

### R-MO rescues spatial reference memory in the hole-board test from negative effects of DMSO

**Fig 3:**
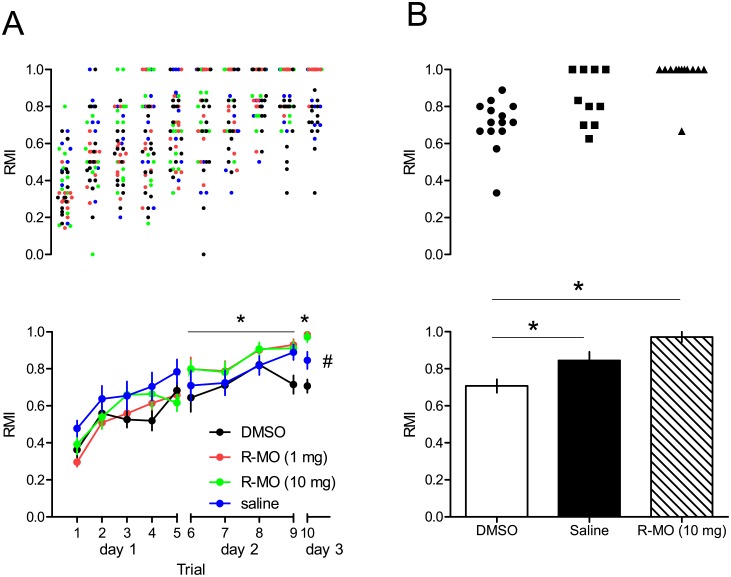
Spatial learning and memory in the Hole-board. The performance was significantly better with improved RMI during day 2 and during retrieval (trial 10) on day 3 of the hole-board testing (A). (B) detailed data presentation for trial 10. Lower panels show the mean ± SEM and the upper panels the individual data points. Two-way repeated measure ANOVA for day 1 & 2 and univariate ANOVA on day 3 were used. * p < 0.05, DMSO vs R-MO 1 & 10 mg/kg. # p < 0.05, DMSO vs saline.

Due to the different phases of training (acquisition at day 1 and day 2 and test of consolidation at day 3) the analysis of the RMI was performed for each day separately. A two–way ANOVA for repeated measures revealed a significant trial effect (F_4,41_ = 24.8, p<0.0001), but no trial X treatment interaction (F_129,129_ = 0.92, p>0.05) and no significant treatment effect (F_3,44_ = 2.38, p>0.05) at day 1. At day 2 a significant trial effect (F_3,42_ = 7.85, p<0.0001), no trial X treatment interaction (F_9,132_ = 0.92, p>0.05) and a significant treatment effect (F_3,44_ = 3.11, p = 0.036) could be detected. Post hoc tests (Dunnett-T, two tailed) revealed a significant difference between the DMSO and R-MO for both 1 mg (p = 0.032) and 10 mg (p = 0.040) dosages with better performance of the two latter groups but not between DMSO and saline (p>0.05). A univariate ANOVA at day 3 results in a significant treatment effect (F_3,44_ = 17.05, p<0.001) and a Scheffé- post hoc test revealed significantly increased memory performance in saline (p = 0.045), and R-MO at 1mg and 10 mg (p<0.001 each) treated compared to DMSO treated rats. Saline treated rats performed not significantly different as compared to R-MO treated rats at 1mg (p = 0.059) and at 10mg (p = 0.095). Data are shown in Fig 3. The differences between groups, although significant are small,especially at day 2. The use of the RMI (ranging from 0 to 1) that corrects the results for individual differences in activity gives little space for group separation. The biological relevance of this behavioral results remained to be proved in further studies.

These differences are not related to neurotoxic effects. There are no statistically significant effects in any test of the neurotoxicity assay (S1F–S1H Fig) or neurological observational battery.

### R-MO rescues negative effects of DMSO in DG synaptic and neuronal plasticity

**Fig 4:**
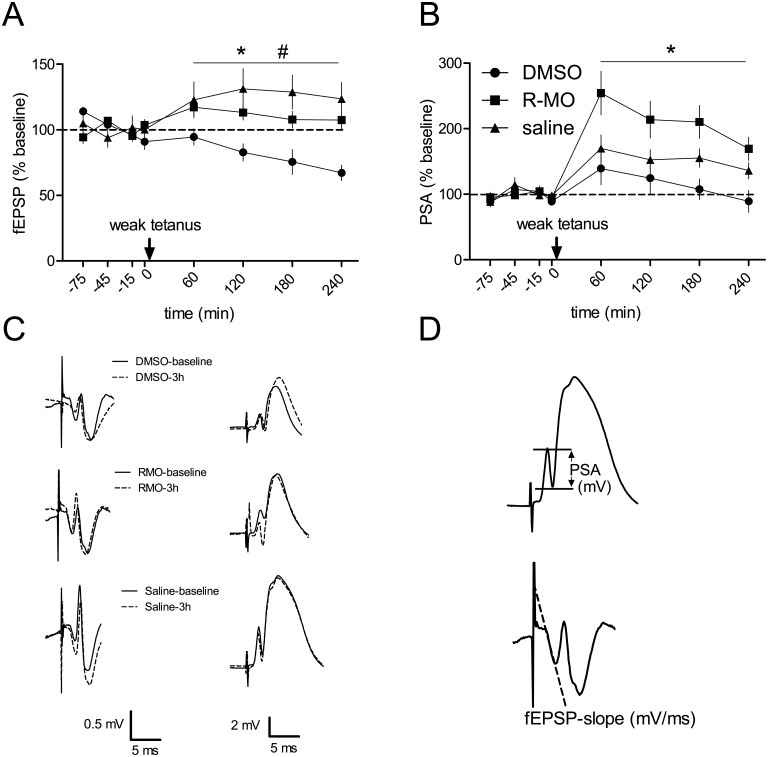
Effect of treatment on field potential measurements in DG: Stimulation with weak tetanus. Data was analyzed with two-way repeated measure ANOVA. Multiple comparison with Dunnett t (2-sided) test reveals difference between groups in fEPSP (A) and in PSA (B) measurements. * dmso vs 10 mg RMO (p<0.05), # saline vs dmso (p<0.05). Graphs shows mean ± SD. (C) Electrophysiological fEPSP (left panel) and PSA (right panel) analogue traces of baseline and 3h post LTP induction are given for DMSO, RMO and saline. (D) Analogue traces of PSA and fEPSP with marks of points of measurement.

The hourly average of post-tetanic fEPSP and PSA measurements were analysed. Two-way repeated measure ANOVA on fEPSP reveals time effect (F_2.2,59.2_ = 2.59, p = 0.076) and no time x treatment interaction (F_4.5,59.3_ = 1.2, p = 0.32) but a significant treatment effect (F_2,26_ = 6.962, p = 0.004). Dunnett-T (2-tailed) post hoc tests revealed increased field potentials for R-MO vs DMSO (p = 0.032) and saline vs DMSO (p = 0.002) treated rats. Comparing the PSA we found a within significant time effect (F_3,102_ = 15.79, p = 0.0001), no time x treatment interaction (F_6,102_ = 1.65, p = 0.14) and a significant treatment effect (F_2,34_ = 4.72, p = 0.015) with Dunnett-T test revealing increased potentials only in R-MO vs DMSO (p = 0.012) but not in saline vs DMSO (p = 0.384) treated rats Fig 4.

Paired pulse induced potentials did not differ between groups at any time point (30 min, 1 h, and 3h) after treatment and no difference in the absence of drugs. I/O curves were not different between the treatment groups (S1I Fig).

### DMSO and R-MO diametrically modulates D1R levels in DG

**Fig 5:**
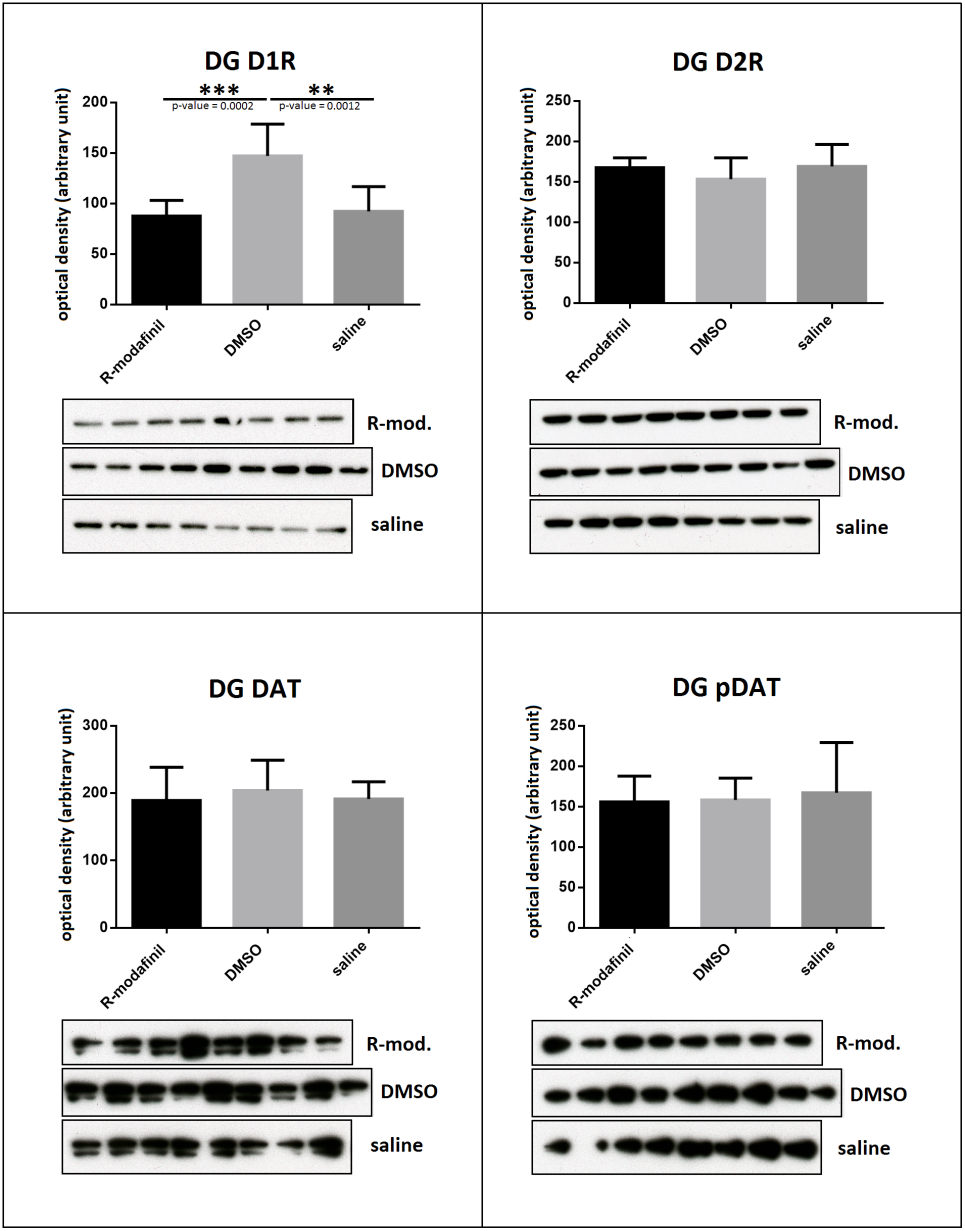
DMSO and R-MO diametrically modulate D1 receptor levels in the dentate gyrus: Synaptosome fraction of the dentate gyrus tissue was analyzed in western blots post LTP induction, probed for D1 (100 kDa), D2 (50 kDa), DAT (115 kDa) and DAT-phospho (75 kDa). D1 levels were significantly increased DMSO treated rats compared to the saline and R-MO (dissolved in DMSO) treated rats. There were no differences in other receptor or transporter levels analyzed. Student’s T-test statistical analysis was performed. Graph indicated mean ± SD. ** p < 0.005, ***p < 0.001.

The synaptosome fraction of the dentate gyrus tissue was analyzed in western blots, probed for D1R, D2R, DAT and ph-DAT. The D1R level was significantly increased in the DMSO treated group compared to the R-MO and saline treated groups (R-MO vs DMSO p = 0.0002, DMSO vs saline p = 0.0012), whereas all other protein levels were similar D2 (R-MO vs DMSO p = 0.184, DMSO vs saline p = 0.237), DAT (R-MO vs DMSO p = 0.285, DMSO vs saline p = 0.181), ph-DAT (R-MO vs DMSO p = 0.875, DMSO vs saline p = 0.703). Thus, since R-MO was dissolved in DMSO, the lack of a difference compared to saline indicate a suppressive effect of R-MO upon DMSO induced upregulation of D1R (Fig 5).

## Discussion

We found parallel effects upon synaptic and neuronal plasticity and memory performance of the wake promoting drug R-MO. The cognition enhancing effect is specific because we found no neurotoxic side effects. This shows that R-MO might have weak cognitive enhancing effects and it suggests molecular mechanisms that might be involved. Spatial memory modulating effects of DMSO have been reported in previous studies, ranging from enhancing effects at low doses [32,33] to impairing effects at high doses [34]. We could observe at least an acutely impairing effect of DMSO on spatial memory and synaptic plasticity, possible long lasting effects, however remain to be investigated.

The wake promoting effects of MO is considered to be partly based on the induced increase of extracellular dopamine [35]. MO induced acute and chronic prolonged wakefulness or sleep deprivation in mice produced LTP at glutamatergic synapses on hypocretin/orexin neurons in the lateral hypothalamus of mice, which could be attenuated by blockade of D1R [36].

In parallel to the dopaminergic effect in the hypothalamus arousal center, systemic administration of MO affects also the other brain regions. MO has an affinity for the DAT in the striatum [37] and induces increased dopamine levels in the prefrontal cortex (PFC) [38] and the caudate nucleus [35]. Further, MO increases the metabolic activity and therefore neuronal activity in the hippocampal sub-regions CA1, CA3 and DG [39]. All these brain regions are critically involved in spatial learning and memory.

However, the effects of MO on synaptic plasticity and spatial memory are different in a brain region and administration protocol dependent manner. Chronic MO treatment impairs PFC dependent learning tasks and PFC-LTP, whereas it decreases reference memory errors in a Olton 4x4 maze, representing the long-term memory and hippocampus dependent component of the task [40].

R-MO has weak effects on memory acquisition, since we found a significant but small increase in RMI on day 2, the main acquisition phase. On day 3 R-MO had no significant effect on memory retrieval above saline whereas the DMSO treatment seemed to have a detrimental effect on both the late learning and the retrieval phase. Increased alertness ore motoric avtivity in R-MO treated rats, that may contribute to the behavioral effects cannot be ruled out, but are unlikely because we could not detect any differences in the neurotoxicity test battery.

Tsanov et al., found better memory acquisition but not consolidation in a water maze spatial task after and during chronic application of MO. The PSA and the fEPSP-LTP was enhanced in the DG of anaesthetized rats, but in the LTP-experiments MO was applied only once before tetanization and a higher dosage (150 mg/kg body weight) was used as during the behavioral experiments (10 and 64 mg/kg bodyweight) [41]. Similar to our study they found the PSA the major component to be affected by MO. The impairing effect of DMSO is bigger for the fEPSP (with smaller effects between groups) then for the population spike, although there is no significant effect of the weak tetanus on PSA. However a EPSP-spike decoupling has often been observed and can be related to different mechanisms [42], such as changes in intrinsic excitability by changes in GABAergic inhibition. Although we did not find differences between the I/O curves after a single administration of DMSO compared to the other groups, the repeated administration of DMSO and R-MO can cause these effects at different extents. GABAergic interneurons mostly synapse on or close to the cell soma [43], such that the firing of granule cells can be changed without affecting the synaptic component and vice versa. Niu et al., found no I/O effects, depressed PSA-LTP but not EPSP-LTP in vivo in morphine treated rats and decreased EPSP-LTP but enhanced PSA-LTP together with impaired spatial learning was found in rats with thyroid hormone insuffiency [44,45]

The drug administration protocol during our LTP-experiments was chosen to match the hole-board experimental design. Possible underlying mechanisms, even dopaminergic, of the effects of MO on synaptic plasticity or spatial memory are unknown and for R-MO only limited information about possible cognitive enhancing and no information about synaptic plasticity effects are available. Therefore we studied these effects in a spatial long-term memory task and upon DG-LTP in combination with molecular markers of dopaminergic mechanisms. We chose a weak tetanic stimulation, inducing early-LTP, because this protocol has been proved to be independent of translation and transcription and thus of the synthesis of proteins [46]. The induction of proteins, possibly including also dopamine receptors, by a stronger stimulation protocol would interfere with the modulation of dopaminergic markers induced by the drug treatment. Early-LTP usually declines to baseline within 4–5 hours, thus an enhancement beyond this time period indicates the establishment of a late-LTP like potentiation.

Unexpectedly, we found increased levels of the D1R levels in the DG in DMSO treated rats. There are some reports that D1R activation can increase dendritic excitability and LTP in the rat DG in a stimulation frequency-dependent manner [47]. Activation of D1R and a mild spike timing depending plasticity stimulation protocol increases the otherwise very narrow time window between presynaptic stimulation and postsynaptic action potentials to increase LTP in the DG [48]. In contrast to this effect of a single stimulation, repeated application (5 times) of the D1R agonist SKF81297 results in a disruption of DG-LTP and long-term recognition memory in mice, whereas a single application maintains plasticity and memory. The impaired LTP and memory after repetitve D1R activation could be rescued by inhibition of the mammalian target of Rapamycin (mTOR) signaling [49]. Specifically the mTOR1 complex has been shown to be activated by cocaine (that also targets the DAT) *via* D1R [50]. Therefore, impaired LTP and memory in DMSO treated rats may be regulated *via* the mTOR pathway mediated by long-lasting enhanced D1R activity. The rescuing effect of R-MO may be based on regulatory functions upon D1R activity. Besides D1R effects on DG-LTP also D2R dependent mechanisms upon DG plasticity have been reported [51]. We did not find D2R and DAT to be regulated, suggesting that R-MO does not act through DAT in the dentate gyrus. Low hippocampal DAT expression have been found in previous studies [52].

However, R-MO may act through DAT in other brain regions with neuromodulatory projections to the hippocampus and thesectseffects probably also involve a regulatory interplay of different dopaminergic receptors and brain regions. Strikingly, we found the hole-board performance and learning reinforced DG-LTP to be also D1R as well as β-adrenergic dependent in a previous study by using the same protocol as in the present [53].

Related to this, there may also be a more indirect β-adrenergic influence on DG-LTP by R-MO. R-MO has indirect sympathomimetic effects, thus activating the sympathetic nervous system and the release of adrenaline and noradrenaline in humans [54]. Due to this effect of R-MO, the release of adrenaline from the adrenal gland is increased which in turn activates vagal afferences to the brain inducing an increased release of noradrenaline in the basolateral amygdala (BLA). Stimulation of the BLA has been shown to enhance DG-LTP and to improve memory [55,56]. Noradrenaline has been shown to be a crucial neuromodulator of DG synaptic plasticity. In humans a 81% increase of urine adrenaline and 33% of noradrenaline has been measured after oral administration of 400 mg MO [54], which may also modulate the activity of the locus coeruleus [57], the main source of noradrenaline in the brain.

Thus, although it remains to be revealed which of these pathways or a multifactorial mechanism is involved in the plasticity and memory rescuing effect of R-MO, pre-synaptic mechanisms are unlikely as suggested by the paired pulse stimulation experiments. We did not find a paired-pulse facilitation, thus a pre-synaptic transmitter release may not be involved in the rescue of synaptic plasticity. Related to a possible increase of pre-synaptic dopamine by R-MO this finding is confirmed by the release assay, not showing an increased release of dopamine by R-MO. We also performed a decisive release assay where we elevated the intracellular sodium concentration by the presence of monensin: this has been shown to unambiguously distinguish inhibitors from releasers [58,59]. The observations from HEK-DAT cells therefore clearly indicate that R-MO does not act as a substrate [60].

We could show that R-MO has cognitive and plasticity modulating effects, though not enhancing but rescuing from deteriorating side effects In addition, some possible pathways of action could be characterized that should be verified in follow up studies.

## Supporting information

S1 File (DOC)

S1 Fig (PDF)
