# Supplementary material for: R-Modafinil exerts weak effects on spatial memory acquisition and dentate gyrus synaptic plasticity
Source: PLoS One. 2017 Jun 23;12(6):e0179675. doi: 10.1371/journal.pone.0179675 (PMC5482457; doi:10.1371/journal.pone.0179675)
Supplement: S1 File — (DOC) [file pone.0179675.s001.doc]

# Supplementary data

# Materials and methods

## Synthesis of (*R*)-Modafinil

R-MO has been synthesized in our laboratory *via* a procedure based on US 7,812,193 B2 patent application. In short, diphenylmethanol was reacted with thiourea to give thiouronium salt, followed by an alkylation with 2-chloroacetamide. Enantio-selective oxidation of R-MO precursor was achieved using a diethyl tartarate titanium (IV) complexes as metal chiral ligands and diisopropylethylamine as a base. The steps for the synthesis are explained here.

**Step one: Synthesis of [(diphenylmethyl)sulfanyl]methanimideamide**

Diphenylmethanol (4.88 g, 0.0265 mol) and thiourea (2.45 g, 0.0322 mol) were dissolved in 20 cm^3^ of water in a round-bottomed flask. The mixture was heated up under reflux to 95°C and 13.8 cm^3^ of hydrobromic acid was gradually introduced over the course of half an hour, upon which the temperature was raised to 107°C. After half an hour of refluxing, the mixture was allowed to cool down to around 80°C and it was subsequently immersed in an ice bath. Crystals of product formed overnight, were filtered on a Hirsch’s funnel, washed with water and dried on a vacuum pump at pressure <1 mbar. 6.33 g of the product was obtained, with the reaction yield of 74%.

**Step two: Synthesis of 2-(benzhydrylthio)acetamide**

[(Diphenylmethyl)sulfanyl]methanimideamide (6.33 g, 0.0196 mol) and potassium –hydroxide (13.52 g, 0.098 mol) were poured in a round-bottomed flask containing 2-choloroacetamide (2.75 g, 0.0294 mol) dissolved in ~ 50 cm^3^ of methanol. The flask was mounted on a magnetic stirrer and the reaction mixture was stirred for two days.

The reaction mixture was condensed under reduced pressure and the residue was suspended in 50 cm^3^ of brine and 50 cm^3^ of ethyl-acetate, shaken vigorously and filtered through a Hirsch’s funnel. Having separated the layers, the rest of the product was extracted from the watery phase with 2 more portions of ethyl-acetate. Organic phases were pooled, dried with anhydrous Na_2_SO_4_, filtered and condensed under reduced pressure. The remaining substance was purified by flash column chromatography on silica gel (5% MeOH in CH_2_Cl_2_ was used as mobile phase), the product was dried on a vacuum pump at pressure <1 mbar. Via this procedure 3.24 g of 2-(benzhydrylthio)acetamide was obtained, with the reaction yield of 64.4%.

**Step three: Synthesis of 2-(*R*)-(benzhydrylsulfinyl)acetamide**

(-)-Diethyltartrate (4.3 cm^3^, 0.0252 mol) and titanium-isopropoxide (3.7 cm^3^, 0.0126 mol) were poured in a round-bottomed flask containing 113.4 μL of water (0.0063 mol) mingled with 24 cm^3^ of acetonitrile. The mixture was stirred and heated for an hour in an oil bath at 50°C and afterwards allowed half an hour to cool down to room temperature. *N,N*-diisopropilethylamine (2.2 cm^3^, 0.0126 mol), 2-(benzhydrylthio)acetamide (3.24 g, 0.0126 mol) and 18 cm^3^ of acetonitrile were added to the mixture, which was stirred for an hour, upon which 2.8 cm^3^ of cumene-hydroperoxide (0.0151 mol) mingled with 6 cm^3^ of acetonitrile was added in a drop-wise fashion and the reaction allowed to proceed for 18 hours.

8 g of ZnSO_4_×7H_2_O was dissolved in 15 cm^3^ of water and added to the reaction mixture. After half an hour of stirring acetonitrile was evaporated, 50 cm^3^ of water and 75 cm^3^ of ethyl-acetate were added to the mixture, which was filtered through a Hirsch’s funnel. Having separated the layers, the remainder of the product was extracted from the water phase with three more portions of ethyl-acetate. Organic phases were pooled, dried with anhydrous Na_2_SO_4_, filtered and condensed under reduced pressure. The remaining substance was purified by flash column chromatography on silica gel (5% methanol in dichloromethane was used as mobile phase) and the product was dried on a vacuum pump at <1 mbar. Upon drying product was additionally purified using preparative-C18 column, on HPLC device, using reversed phase conditions. Solid material was dissolved in methanol at concentration of 100 mg per ml. 0.5 ml of solution was applied on preparative 21.2 mm i.d. x 25 cm Zorbax SB-C18 (Agilent Technologies, United States) column per each run on a Shimadzu LC-8A HPLC system (Shimadzu Corporation, Tokyo, Japan) using gradient of acetonitrile in water (30-50% in 10 min, 100% ACN 10-15 min) and the procedure was repeated until the whole amount of material was purified. Purified fractions were pooled together and concentrated on a rotary evaporator and the final product was dried on a vacuum pump at <1 mbar.

Via this procedure 1.1 g of (*R*)-modafinil was obtained (stoichiometrical yield of the reaction was 32%). The overall purity of the compound, determined by C18 analytical column based method using reversed-phase conditions was 98.48% (Figure S1). Enantiomeric purity of the compound, determined by HPLC based method using chiral column was 96.23% (Figure S2).

MS. [M+Na^+^]= 296.0714 (Figure S3).

^1^H NMR (600 MHz, DMSO-d_6_, 23°C): δ = 3.22, 3.36 (AB, *^2^J* = 13.5, 2H, C*H*_2_), 5.34 (s, 1H, C*H*), 7.67 (s, br, 1H, N*H*), 7.53-7.49 (m, 2H, *H*-4 phenyls), 7.39-7.44 (m, 4H, *H*-3,5 phenyls), 7.30-7.38 (m, 4H, *H*-2,6 phenyls), 7.32 (s, br, 1H, N*H*); ^13^C{^1^H}NMR (150 MHz, DMSO-d_6_, 23°C): δ = 56.17 (*C*H_2_), 68.79 (*C*H), 127.95 (*C*-4, phenyl), 127.98 (*C*-4, phenyl), 128.51 (2x *C*-3,5, phenyls), 129.06 (*C*-2,6, phenyl), 129.73 (*C*-2,6, phenyl), 134.95 (*C*_q_-1, phenyl), 137.21 (*C*_q_-1, phenyl), 166.39 (*C*ONH_2_). (Figure S4)

^13^C{^1^H}NMR (150 MHz, DMSO-d_6_, 23°C): δ = 56.17 (*C*H_2_), 68.79 (*C*H), 127.95 (*C*-4, phenyl), 127.98 (*C*-4, phenyl), 128.51 (2x *C*-3,5, phenyls), 129.06 (*C*-2,6, phenyl), 129.73 (*C*-2,6, phenyl), 134.95 (*C*_q_-1, phenyl), 137.21 (*C*_q_-1, phenyl), 166.39 (*C*ONH_2_). (Figure S5)

**Abbreviations:**

Solvents and reagents: MeOH, methanol; CH_2_Cl_2_, dichloromethane; ACN, acetonitrile.

NMR characterization: br, broad; multiplicity: m, multiplet; s, singlet, d, doublet; t, triplet; q, quaternary or quartet; qu, quintet; sept, septet; AA’BB’: the four most intense peaks are given; AA’ or BB’ part of AA’BB’; m[e.g. t, dd, q, tt, sext] notation in brackets describes the overall appearance of the signal pattern;

DMSO-d_6_, hexadeuterio dimethyl sulfoxide (CAS RN 2206-27-1); HSQC, Heteronuclear Single Quantum Coherence; HMBC, Heteronuclear Mutliple Bond Correlation; COSY, Correlation Spectroscopy.

**Analytical Characterization (NMR, Mass Spectrometry, HPLC)**

NMR spectra were recorded on Bruker Avance 600-MHz spectrometers (NMR Centre, Faculty of Chemistry, University of Vienna). The software used for processing of 1D- (^1^H, ^13^C) and 2D- (COSY, HMBC, HSQC) NMR spectra was SpinWorks 4.2.4 (copyright 2010, Kirk Marat, University of Manitoba). Coupling constants (*J*) are given in Hertz (Hz) and refer to the first order interpretation (apparent coupling constants Japp are provided).Assignment of resonances was performed with COSY, HSQC and HMBC, respectively. Solvents used for NMR spectroscopy DMSO-d_6_, hexadeuterio dimethyl sulfoxide (CAS RN 2206-27-1) was stored over a molecular sieve (4 Å). 2D NMR techniques used for the assignment of 1H and 13C resonance signals: HSQC, HMBC and COSY. Chemical shift calibration: CDCl_3_, ^1^H δ = 7.26, ^13^C δ = 77.16; DMSO-d_6_, ^1^H δ = 2.50, ^13^C δ = 39.52 ppm (H. E. Gottlieb, V. Kotlyar, A. Nudelman. NMR Chemical Shifts of Common Laboratory Solvents as Trace Impurities.*J. Org. Chem.* 1997, 62, 7512-7515).

HRESIMS spectra were obtained on a maXis HD ESI-Qq-TOF mass spectrometer (Bruker Daltonics, Bremen, Germany). Samples were dissolved to 20 µg/mL in MeOHand directly infused into the ESI source at a flow rate of 3 µL/min with a syringe pump. The ESI ion source was operated as follows: capillary voltage: 0.9 to 4.0 kV (individually optimized), nebulizer: 0.4 bar (N_2_), dry gas flow: 4 L/min (N_2_), and dry temperature: 200 °C. Mass spectra were recorded in the range of *m/z* 50 – 1550 in the positive-ion mode. The sum formulas were determined using Bruker Compass DataAnalysis 4.2 based on the mass accuracy (Δ*m/z* ≤ 2 ppm) and isotopic pattern matching (SmartFormula algorithm).

HPLC spectra were obtained on LC-2010A HT Liquid Chromatograph device (Shimadzu Corporation, Tokyo, Japan). R-Modafinil was dissolved in MeOH at concentration of 1 mg/ml. 10 µl of the sample was applied on Acclaim™ 120 C18 columns 2.1x150 mm (ThermoScientific, Massachusetts, United States) under reversed-phase conditions (10-90% gradient of acetonitrile in water).

For determining enantiomeric purity of the R-Modafinil, HPLC measurements were performed on LC-2010A HT Liquid Chromatograph device (Shimadzu Corporation, Tokyo, Japan). R-Modafinil was dissolved in MeOH at concentration of 1 mg/ml. 10 µl of the sample was applied on Chiralpack IA column 4.6x150 mm (Daicel Corporation, Tokyo, Japan). 100% ACN was used as mobile phase.

## Neurological toxicity assays

**Elevated plus maze**

The elevated plus maze tests the anxiety in rodents, it consists of plus shaped plastic lanes (10 cm in width, 1.10 m in length placed at an elevation of 62 cm from the ground), with one lane surrounded by black plastic walls (40 cm in height) (closed arm), the other arm has no walls (open arm), The arms were connected by 10 x 10 cm central open area, in which the animals were placed in and allowed to explore the maze for 5 min. The movements were recorded by a tracking system and stored on a computer. The time (s) the animals spent in the closed and the open arms were considered for the analysis. The parameters that were measured were open arm leaving (n), closed arm leaving (n), total distance covered (m), distance in open arm (m), distance in closed arm (m), time spent in open arm (s), time spent in closed arm (s), and resting time (s) similar as in (Weitzdoerfer et al., 2004).

**Open field**

The open field consists of a black plastic board (1.20 m x 1.20 m) surrounded by black plastic walls (50 cm in height). The rats were placed in the arena and allowed to explore it for 10 min. The movements were recorded by a video tracking system and stored on a computer. Standard parameters for locomotor activity i.e. the total distance covered (m), resting time (s), amount of local movement (%), amount of large movement (%), average velocity (m/s), number of times crossing the center, frequency of spontaneous changes of direction, and time spent in the corners (s). (Weitzdoerfer et al., 2004).

**Rota rod**

The rota rod (Rota Rod ‘‘Economex’’, Columbus Instruments, Ohio, USA) tests balance and coordination of rats. It consists of a rotating plastic cylindrical barrel which accelerates from 4 to 40 rpm over the course of 5 min. The rats were placed on the barrel and the time it can hold the barrel without falling was recorded. Each rat received three pre-training trials followed by three consecutive trials and the longest time it can hold the barrel was used for analysis.

**Neurological observational battery of tests**

The following parameters were measured in neurological observational battery. Body Position, Palpebral closure, Locomotor activity, Bizarre behavior, Exophthalmos, Respiratory rate, Tremors, Twitches, Convulsions, Transfer arousal (appearance), Spatial locomotion, Startle response, Piloerection, Gait, Limb rotation, Pelvic elevation, Tail elevation, Finger approach, Finger withdrawal, Touch escape, Positional passivity, Visual placing, Grip strength, Body tone, Pinna (ear stimulation), Cornea, Toe pinch, Wire maneuver, Skin color, Diarrhea, Limb tone, Provoked biting, Tail pinch and Righting reflex.

**Forced swim test**

The rats were placed in a translucent plastic cylinder (20 cm in diameter and 45 cm in height filled with water up to a 33 cm level). The test consists of training and a test session on two consecutive days. The rats were placed in a translucent plastic cylinder (20 cm in diameter and 45 cm in height filled with water up to a 33 cm level at a temperature of 25 °C). The rats were placed in the cylinder for 10 min during the training and 5 min during the test session. Movements in the cylinder were recorded by a video tracking system and stored on a computer. The time the animals spent immobile was used for the analysis.

**References**

Weitzdoerfer, R., Pollak, A., Lubec, B., 2004. Perinatal asphyxia in the rat has lifelong effects on morphology, cognitive functions, and behavior. Semin. Perinatol. 28, 249–256.
