# Supplementary material for: R-Modafinil exerts weak effects on spatial memory acquisition and dentate gyrus synaptic plasticity
Source: PLoS One. 2017 Jun 23;12(6):e0179675. doi: 10.1371/journal.pone.0179675 (PMC5482457; doi:10.1371/journal.pone.0179675)
Supplement: S1 Fig — (PDF) [file pone.0179675.s002.pdf]

A

Purity of R-Modafinil determined by HPLC

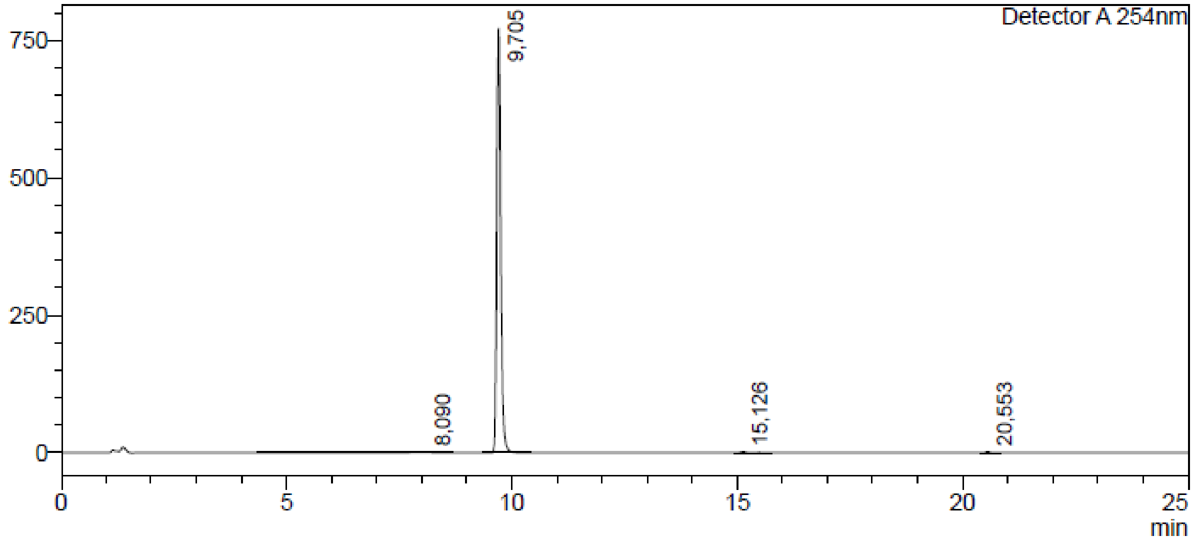

Peak#	Ret. Time	Area	Conc.	Area%
1	8,090	35725	0,748	0,748
2	9,705	4706334	98,483	98,483
3	15,126	22366	0,468	0,468
4	20,553	14423	0,302	0,302
Total		4778848		100,000

B

Enantiomeric purity of R-Modafinil

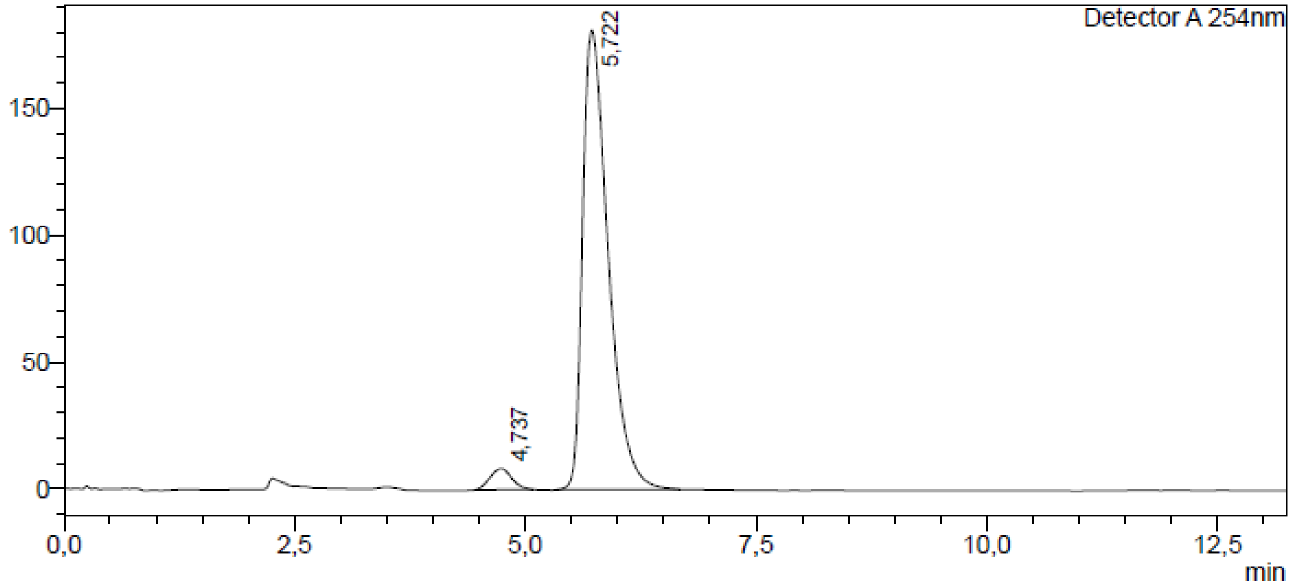

Peak#	Ret. Time	Area	Conc.	Area%
1	4,737	136980	3,767	3,767
2	5,722	3499330	96,233	96,233
Total		3636310		100,000

C

High-res Mass Spectra of R-Modafinil

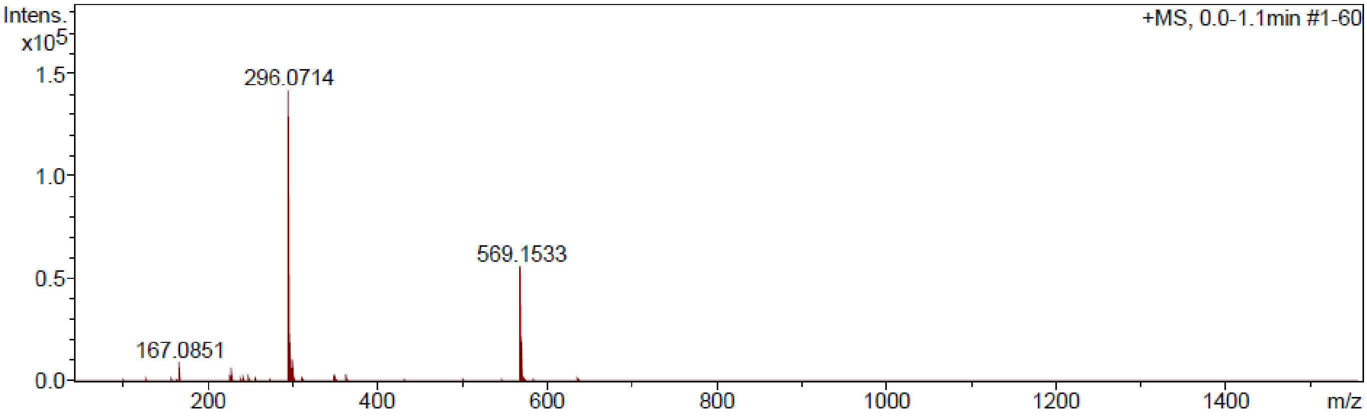

Meas. m/z	#	Ion Formula	m/z	err [ppm]	mSigma	# mSigma	Score	rdb	e⁻ Conf	N-Rule
296.0714	1	C15H15NNaO2S	296.0716	0.6	12.2	1	100.00	8.5	even	ok

D

H NMR Spectra of R-Modafinil

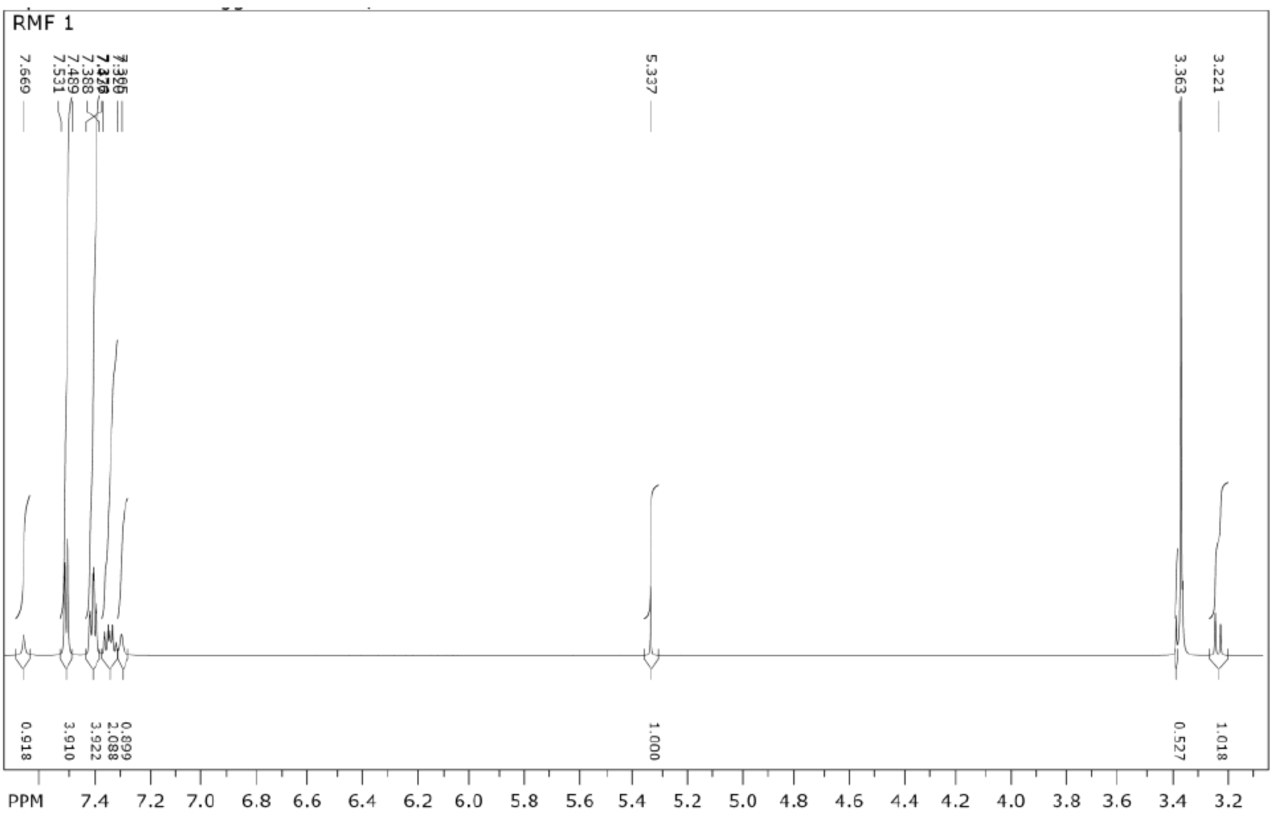

E

C NMR Spectra of R-Modafinil

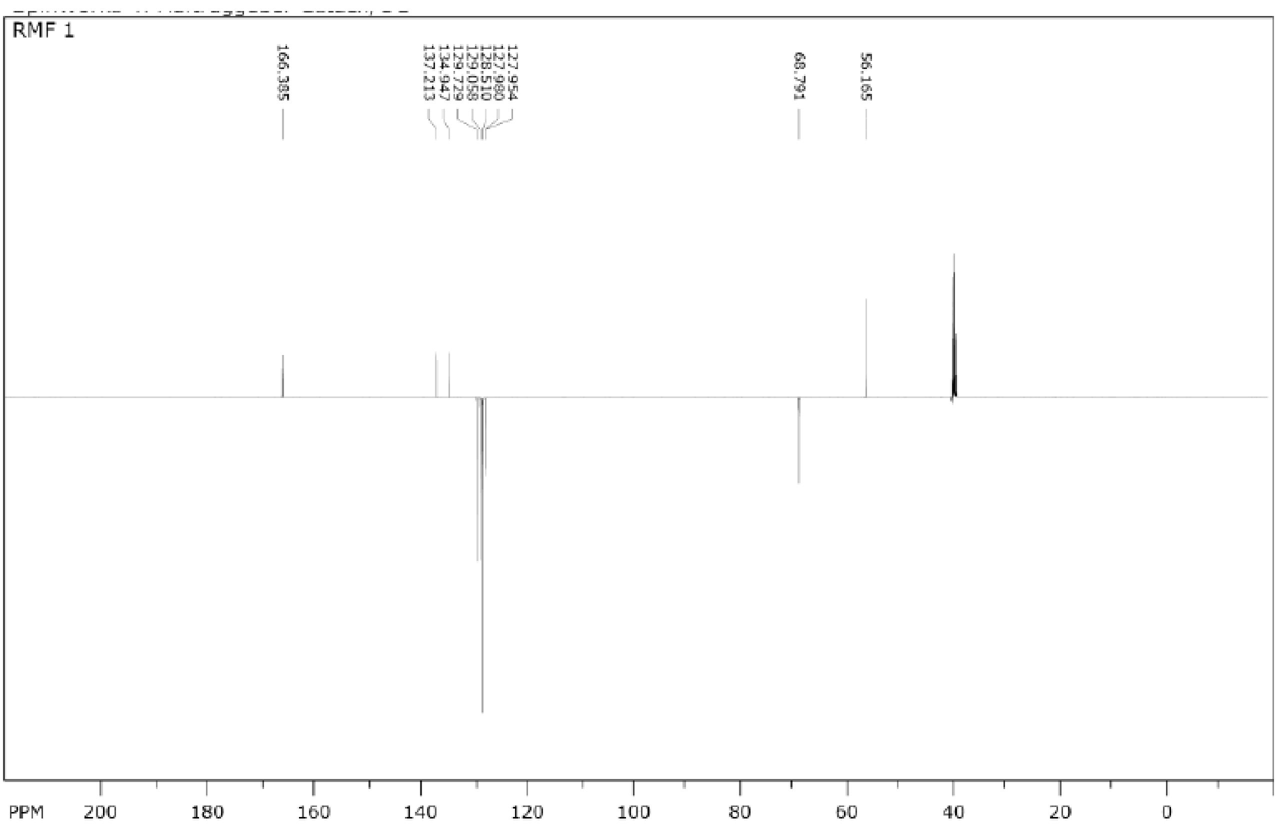

F

Elevated plus maze

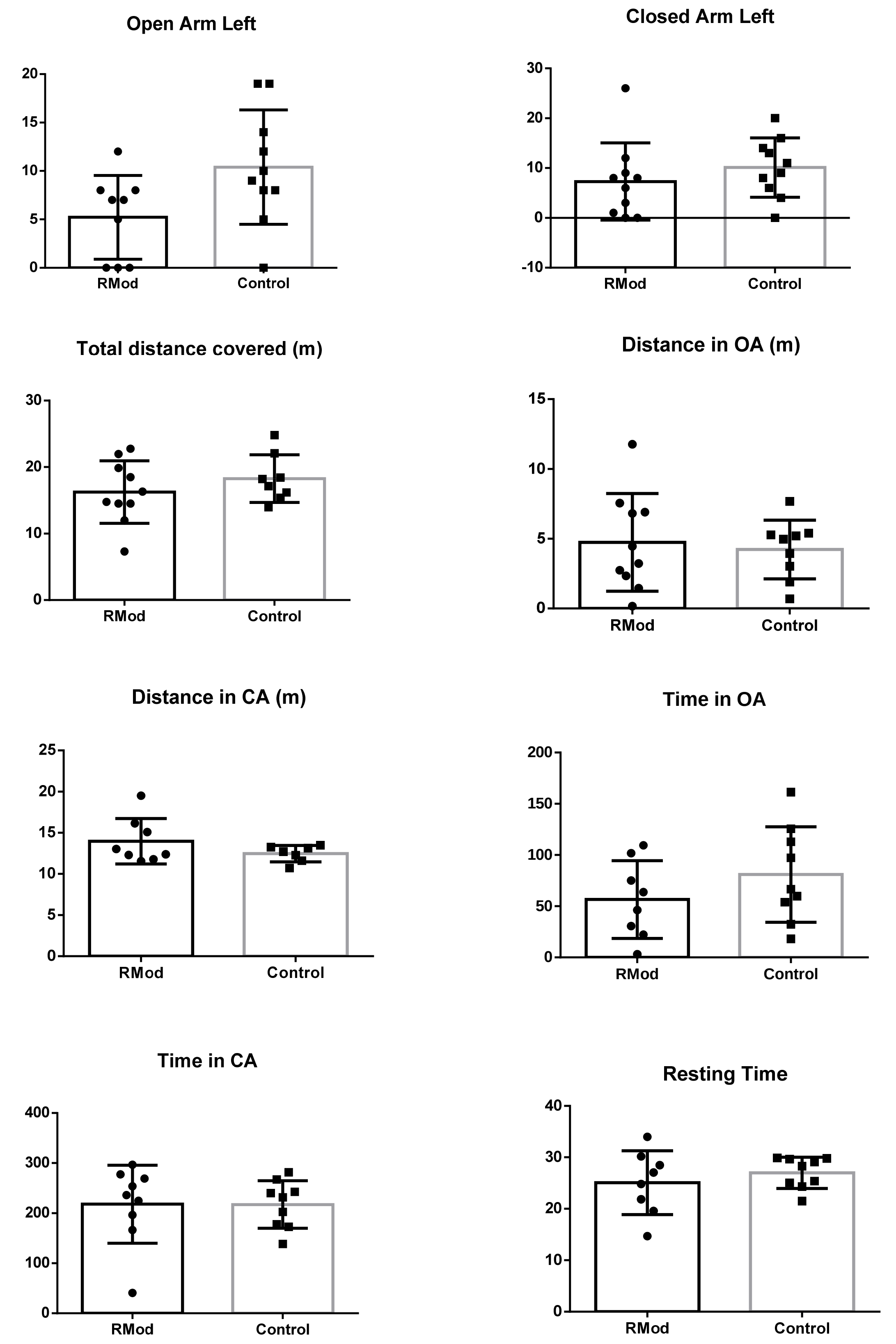

Elevated plus maze: Rmod – R-Modafinil 10mg/kg, control – DMSO, OA – Open arm, CA – Closed arm, graphs shows mean ± SD

G

Rota rod

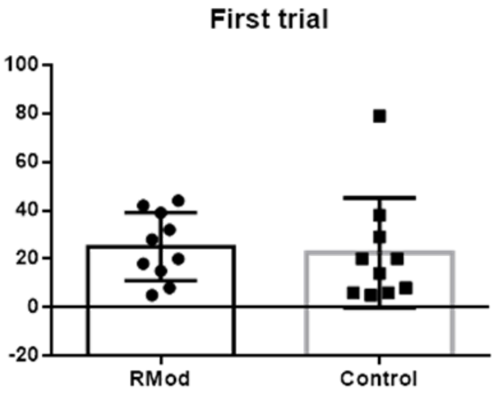

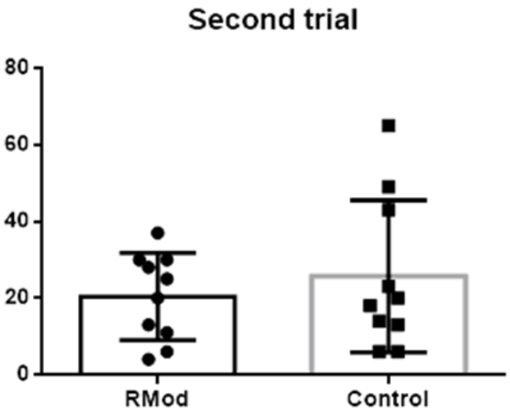

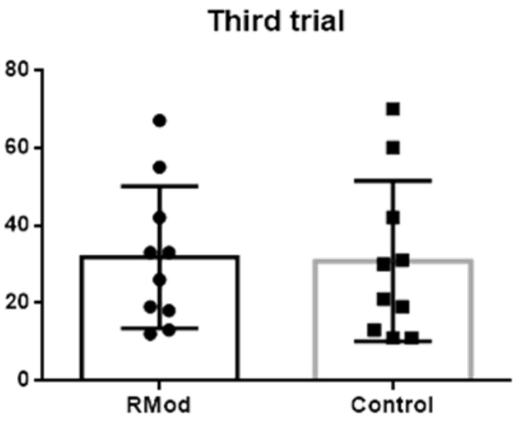

Forced swim test

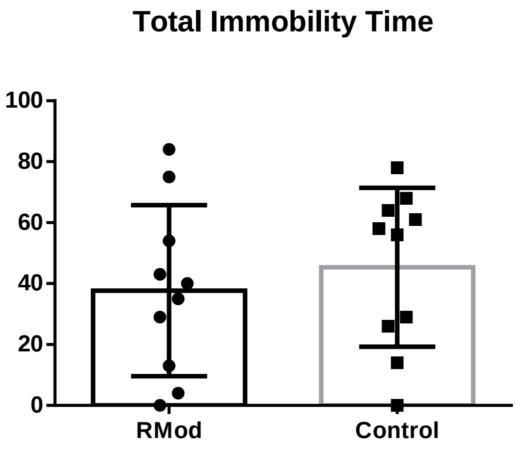

RMod – R-Modafinil 10mg/kg, control – DMSO, graphs shows mean ± SD

H

Open field test

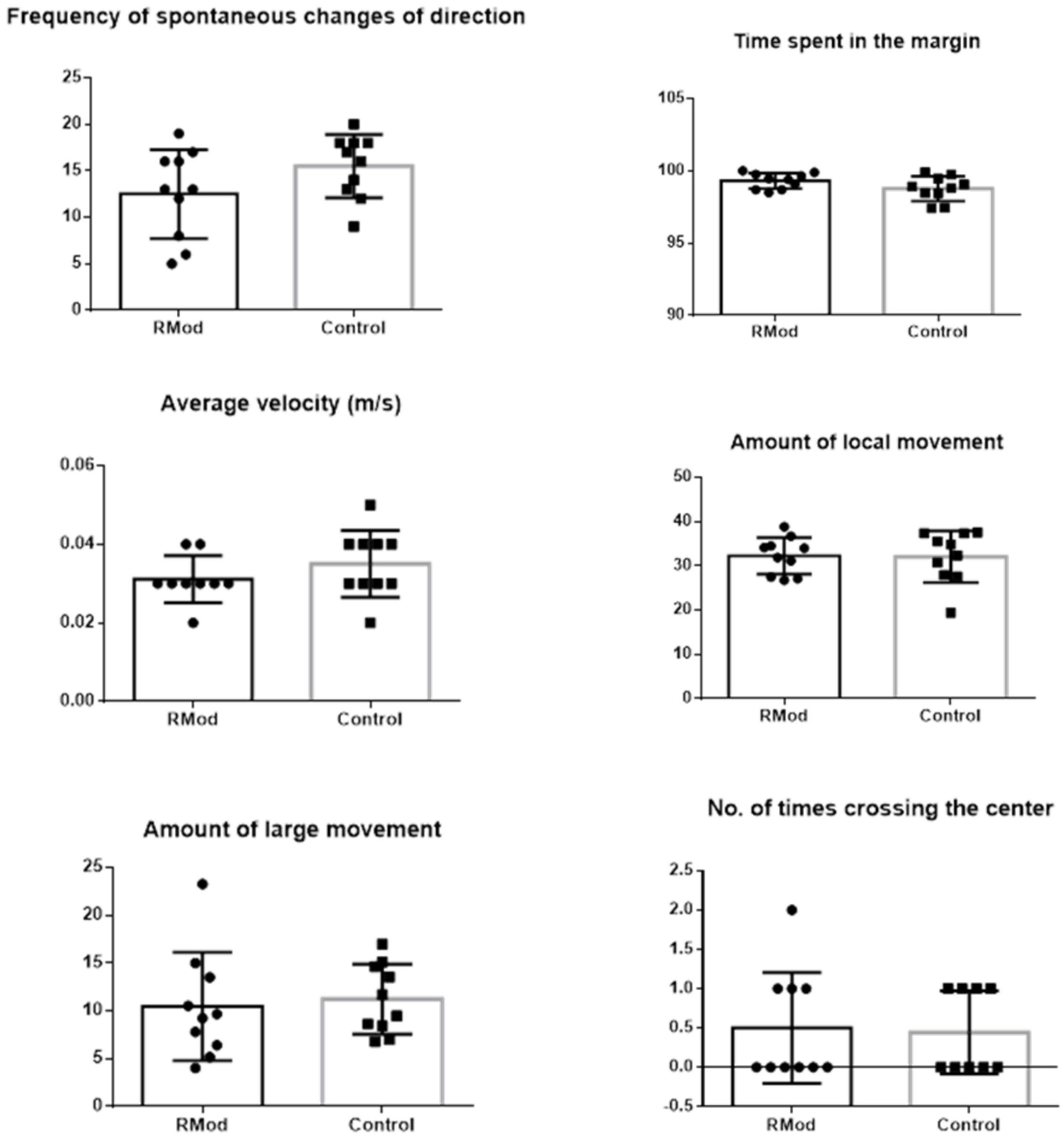

Open field test: Rmod – R-Modafinil 10mg/kg, control – DMSO, graphs shows mean ± SD

I

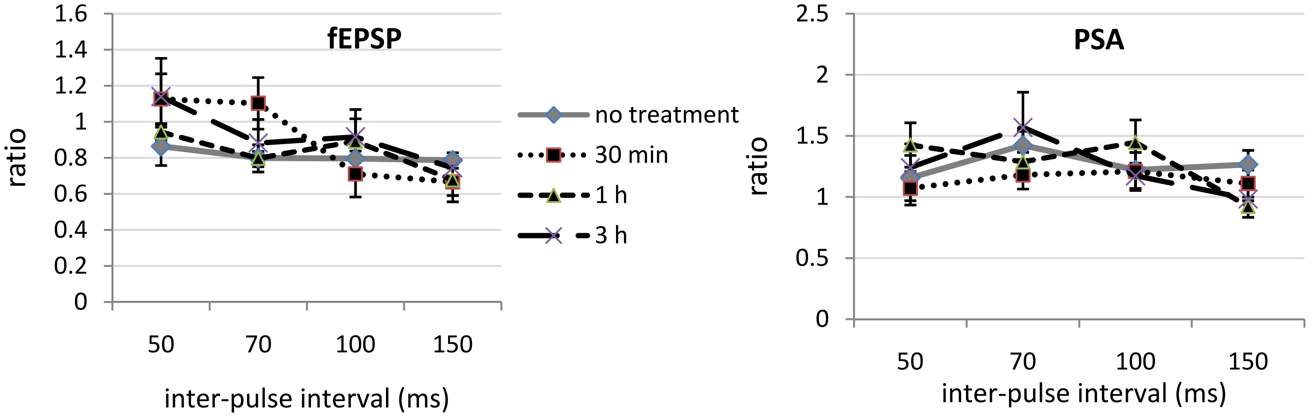

Effect on Paired pulse facilitation: Paired pulse facilitation: Paired pulse stimulation without treatment and 30 min, 1 h and 3 h post administration of R-MO 10mg/kg revealed no differences between groups. Graph shows mean ± SD
